# Early warning of systemic risk in stock market based on EEMD-LSTM

**DOI:** 10.1371/journal.pone.0300741

**Published:** 2024-05-21

**Authors:** Meng Ran, Zhenpeng Tang, Yuhang Chen, Zhiqi Wang

**Affiliations:** 1 School of Management, Fujian University of Technology, Fuzhou, China; 2 Fujian Agriculture and Forestry University, Fuzhou, China; 3 Quanzhou Branch of China Banking and Insurance Regulatory Commission, Quanzhou, China; University 20 Aout 1955 skikda, Algeria, ALGERIA

## Abstract

With the increasing importance of the stock market, it is of great practical significance to accurately describe the systemic risk of the stock market and conduct more accurate early warning research on it. However, the existing research on the systemic risk of the stock market lacks multi-dimensional factors, and there is still room for improvement in the forecasting model. Therefore, to further measure the systemic risk profile of the Chinese stock market, establish a risk early warning system suitable for the Chinese stock market, and improve the risk management awareness of investors and regulators. This paper proposes a combination model of EEMD-LSTM, which can describe the complex nonlinear interaction. Firstly, 35 stock market systemic risk indicators are selected from the perspectives of macroeconomic operation, market cross-contagion and the stock market itself to build a comprehensive indicator system that conforms to the reality of China. Furthermore, based on TEI@I complex system methodology, an EEMD-LSTM model is proposed. The EEMD method is adopted to decompose the composite index sequence into intrinsic mode function components (IMF) of different scales and one trend term. Then the LSTM algorithm is used to predicted and model the decomposed sub-sequences. Finally, the forecast result of the composite index is obtained through integration. The empirical results show that the stock market systemic risk index constructed in this paper can effectively identify important risk events within the sample period. In addition, compared with the benchmark model, the EEMD-LSTM model constructed in this paper shows a stronger early warning ability for systemic financial risks in the stock market.

## 1. Introduction

The systemic risk of the stock market has always been the focus of attention of governments and regulators. The plunge of the Chinese market in 2015 and the impact of the trade war in 2018 led to a short-term plunge of the Shanghai and Shenzhen stock markets across the board, which highlighted the severity of the systemic risk [1]. Due to the influence of multiple internal and external factors, the systemic risk of the stock market has been accumulating and may trigger a crisis at a certain level [2]. The rapid development of China’s stock market and its importance in the national economy make the establishment of a scientific and effective systemic risk early warning indicator system especially critical to ensure the orderly development and protect the stability of the financial system and investor rights and interests. At present, there are deficiencies in the study of systemic risk in the stock market. On the one hand, the qualitative research on stock market systemic risk mainly focuses on banking systemic risk, which has not attracted enough attention until after the subprime crisis [3]. On the other hand, existing systemic risk early warning models are mostly based on a single artificial intelligence algorithm, which cannot fully consider the complex multi-scale nature of the stock market, resulting in limited early warning accuracy [4]. In terms of behavioral factors, traditional models often ignore the important impact of investor sentiment on market trends, while the actual economic activities of these factors have an impact on future market trends, especially in the Chinese stock market is more obvious [5]. Therefore, the study firstly selects 35 stock market systematic risk indicators from three perspectives: macroeconomic operation, market cross-infection and stock market itself, and constructs a comprehensive stock market systematic risk indicator system in line with the actual situation in China. In addition, the EEMD decomposition and integration technique in the TEI@I complex system research methodology is also borrowed. Combined with the current research results of artificial intelligence and deep learning, the EEMD-LSTM combination model is constructed to provide early warning of systemic risk in the Chinese stock market.

The study has two major contributions: first, a more comprehensive stock market systemic risk composite index is created, which takes into account multidimensional complex factors, such as investor sentiment, and describes stock market systemic risk more accurately compared to previous early warning studies with one-dimensional information. Second, the TEI@I complex system research methodology is used to propose the EEMD-LSTM model, which takes into account complex nonlinear interactions and improves early warning accuracy by decomposing and predicting the characteristic pattern components and trend terms at different scales. The study also makes three contributions in terms of economic rationality: first, the EEMD-LSTM framework enables the financial time series to be split to provide a clear view of the data analysis, and the LSTM model is effective in predicting future changes in the stock market. Second, the validity of the model in practice is verified through data visualization by combining the data of mergers and acquisitions of various industries in the Chinese stock market from 2007 to 2019. Third, the economic significance of the model for investors, regulators and market stability in the Chinese stock market is discussed through result testing. These contributions highlight the research in terms of practicality and potential value to stock market related stakeholders.

## 2. Literature review

At present, research on systemic risk early warning models is mainly divided into discrete early warning models and continuous early warning models. Traditional discrete systemic risk early warning models mainly include FR probability model [6], STV cross-sectional regression model [7] and KLR model [8]. The same characteristics of the above model are as follows. The meaning of systemic crisis is pre-defined, with 0 and 1 chosen as the dependent variables. Non crisis and crisis situations are defined, and warning indicators are selected as independent variables to construct regression equations. The correlation between economic variables and crises is analyzed. A set of optimal warning indicators is used to determine the likelihood of a systemic crisis outbreak. The above early warning models have the following shortcomings. Firstly, the definition and threshold of crisis are set subjectively, which has the disadvantages of “man-made crisis”. Secondly, continuous financial stress variables are discretized, which can easily lead to information loss. Thirdly, traditional static linear models have defects in capturing the dynamics before and after the crises, which cannot depict the transitions between different states [9]. In recent years, many scholars try to break through the framework of the discrete early warning system and build a continuous early warning system. Compared with the early discrete early warning system, the financial stress index (FSI) constructed by Illing and Liu can be regarded as a relatively successful attempt in the continuous early warning system [10]. Different from the 0–1 discrete variable method, the financial stress index is a continuous measurement indicator that presents the changing trend and severity of risk pressure changes in the financial system in a time series manner. This has advantages in revealing and warning potential systemic risks. Gao selected the stress index system mainly from the perspective of countercyclical capital regulation [11]. Zhang et al. clarified the stress index system from four dimensions, i.e., the financial policy system, macroeconomic environment, traditional banking, and shadow banking when studying the systemic risk [12]. Yang et al. set up the index system mainly from three perspectives, i.e., asset bubbles, bank crisis and currency crisis [13]. It can be seen that there is no unified systemic risk index at present, and the research on systemic risk early warning in stock market is even less. Most of the existing studies are based on specific one-dimensional information. Ruan explored the correlation between the investment behavior of individual investors and the healthy development of the stock market in the real stock market investment environment from the perspective of behavioral finance [14]. Zhou inspected the impact of institutional investors’ behavior on the stock market during the China stock market bubble, and found that institutional investors did not eliminate the price deviation, but further promoted the emergence and expansion of the stock market bubble [15]. This paper draws on the existing research findings and takes multiple factors such as peripheral market data, macroeconomic data, other financial market data, stock market trading data, investor behavior factors, etc. into comprehensive consideration to build a composite index of the stock market systemic risk for measuring the systemic risk status of the Chinese stock market, and early warning of the stock market systemic risk is given by forecasting the future trend of the stock market systemic risk composite index.

Similarly, scholars have conducted extensive research on time series prediction problems. Table 1 lists recent relevant research papers and elaborates on the models and contributions of these papers.

**Table 1:** Recent papers on time series prediction.

Reference	Forecast Model	Application Scenario	Main Contribution and Novelty
Henrique et al. [16]	SVM	Stock Market	The study summarizes existing literature. The SVM and ANN are the most commonly used tools in financial market forecasting analysis and modeling
	ANN		
Yoshihara et al. [17]	RNN	Stock Market	The study proposes a recursive deep neural network based market trend prediction method.
Khattak et al. [18]	LASSO	Stock Market	The proposed LASSO algorithm has more advantages compared to traditional regression methods.
Xiong et al. [19]	LSTM	Stock Market	The study proves that the prediction accuracy of the model has significantly improved.
Liu [20]	LSTM	Stock Market	The proposed LSTM performs better than traditional models in predicting volatility over longer intervals.
Bukhari et al. [21]	ARFIMA-LSTM	Financial Market	The study proposes a new hybrid recurrent network.
Wang [22]	TEI@I	Crude Oil Market	The study proposes a new price prediction method.
Wu and Huang [23]	EEMD	Southern Oscillation Index	The study proposes EEMD for the first time to improve the susceptibility to modal aliasing in EMD decomposition.
Yang and Yang [24]	BRR-EEMD	Wind Speed	The study proposes a mixed wind speed prediction method and achieves good prediction results.
Liu et al. [25]	EEMD-BP EEMD-VM	Urban Water Consumption	The proposed combination model has better predictive performance than traditional models.

So far, scholars have conducted extensive researches on time series forecasting with three major methods: traditional econometric model, artificial intelligence (AI) prediction and combining models. Traditional econometric models, such as ARMA model, ARIMA model, GARCH model and vector autoregression model (VAR), have much limitations in dealing with nonlinear and non-stationary time series [26]. To address these issues, AI uses machine learning technology to train historical data, to realize higher prediction accuracy in nonlinear time series data. Typical models include Artificial Neural Network (ANN), Genetic Algorithm (GA) and Support Vector Machine (SVM). However, traditional AI models with limited accuracy cannot effectively demonstrate the correlation between data before and that after time series. However, with the further development of deep learning technology, Recurrent Neural Network (RNN) gains more popularity for its embedded feedback and cyclic structure. It can handle the auto-correlation characteristics of time series data. However, using backpropagation through time algorithms in RNN can lead to gradient vanishing or exploding. Moreover, long-term dependence cannot be better resolved. To solve these problems, LSTM, as a RNN variant, also overcomes the gradient vanishing and explosion problems in RNN network [27, 28]. Therefore, LSTM has huge advantages in processing time series with long time spans and sequence dependencies [29].

Although artificial intelligence models perform well in predicting highly complex data, they are sensitive to parameter and model settings. Local minima and over fitting are prone to occur. In summary, a single model that fits all situations is not feasible. Therefore, a third prediction method has emerged, which is the combination model, typically TEI@I methodology for complex system research that proposed by Wang et al. [30]. This methodology utilizes the idea of "decomposition-integration". More specifically, firstly, a complex system is decomposed, and then dealt with by AI method in non-linearity and high complexity. Next, the integration approach is adopted to integrate those decomposed complex system for overall analysis and modeling. Moreover, pre-processing is conducted to decompose the original time series into different subsequences. Due to their different properties, each sub-sequence needs separate modeling and prediction. By combining all the prediction results of individual sub-sequence, higher accuracy can be realized. In terms of pre-processing, typical methods include Wavelet Decomposition (WD) [31] and Empirical Mode Decomposition (EMD) [32]. In particular, the EMD algorithm decomposes data based on the time scale characteristics of different frequencies, rather than relying on any base function. In this way, it is highly different from the WD method and far superior to the WD method in processing non-stationary and nonlinear data. Therefore, this paper chooses the EMD model to decompose the original time series. Furthermore, to improve mode mixing problem in EMD decomposition, Wu et al. proposed the Ensemble Empirical Mode Decomposition (EEMD) algorithm. This algorithm helps extract true modal components by adding white noise to the original temporal data. Through multiple averaging, the noise cancels out each other, thus effectively avoiding mode mixing.

In summary, this paper constructs a comprehensive index of systemic risk in the Chinese stock market to measure systemic risk in the stock market. It is expected to provide early warning for systemic risk in the stock market by predicting the comprehensive index future trend of systemic risk. Referring to the TEI@I methodology for complex system research, the EEMD-LSTM model is proposed. The EEMD algorithm is used to decompose the original sequence of the stock market systemic risk comprehensive index. Artificial intelligence algorithms such as LSTM are used to predict and model each sub sequence. Finally, all these analyses are integrated to gain the overall prediction results with higher accuracy and more efficient warning in the stock market.

## 3. Methodology

### 3.1 Ensemble empirical mode decomposition

To solve mode mixing in EMD, Wu and Huang proposed the EEMD algorithm, which adds the minimum amplitude white noise sequence to the original time series. The specific steps for its decomposition are as follows:

1) Adding multiple times white noise sequences of equal length *n*_*i*_(*t*) that satisfy normal distribution in the original time series *x*(*t*), namely:
$$x_{i}(t)=x(t)+n_{i}(t)$$ (1)
  In Eq (1), *x*_*i*_(*t*) is the newly generated time series after adding white noise for the *i*-th time.
2) Conducting EMD decomposition for the newly generated time series to obtain different IMF components *c*_*i*,*j*_(*t*) and residual terms *r*_*i*_(*t*). *c*_*i*,*j*_(*t*) is the *j*-th IMF component obtained from EMD decomposition after adding white noise for the *i*-th time.
$$x_{i}(t)=\sum_{j=1}^{J}C_{i,j}(t)+r_{i}(t)$$ (2)
3) Continuously repeating steps (1) and (2). The original time series is repeatedly supplemented with white noise signals to obtain the final decomposition sequence of different IMF modal components:
$$C_{j}(t)=\frac{1}{N}\sum_{i=1}^{N}C_{i,j}(t)$$ (3)
  In Eq (3), *c*_*j*_(*t*) is the *j*-th IMF component obtained after EEMD decomposition. *N* is the amount of white noise sequences.
4) Obtaining the final EEMD decomposition sequence, namely:
$$x(t)=\sum_{j=1}^{J}C_{j}(t)+r(t)$$ (4)
  In Eq (4), *c*_*j*_(*t*) is components arranged in each frequency band from high to low frequency in the initial sequence. *r*(*t*) is the residual term independent of each component.

### 3.2 Recurrent neural network

In traditional fully-connected networks, the signals from each layer of neurons can only be transmitted to the next layer. The training samples are independent at different times, thus being unable to characterize the temporal dependencies in time series. Unlike traditional multi-layer perceptrons, Recurrent Neural Network (RNN) is affected by the temporal order of samples, which can effectively handle above problems [33].

The main logic behind RNN is that the current sequence output depends on the current and previous inputs. The RNN network stores previous information and applies it to the current output calculation. It means that the nodes between hidden layers are no longer disconnected. Moreover, the input of the hidden layer includes the current input and the previous output of the hidden layer [34].

**Fig 1:**
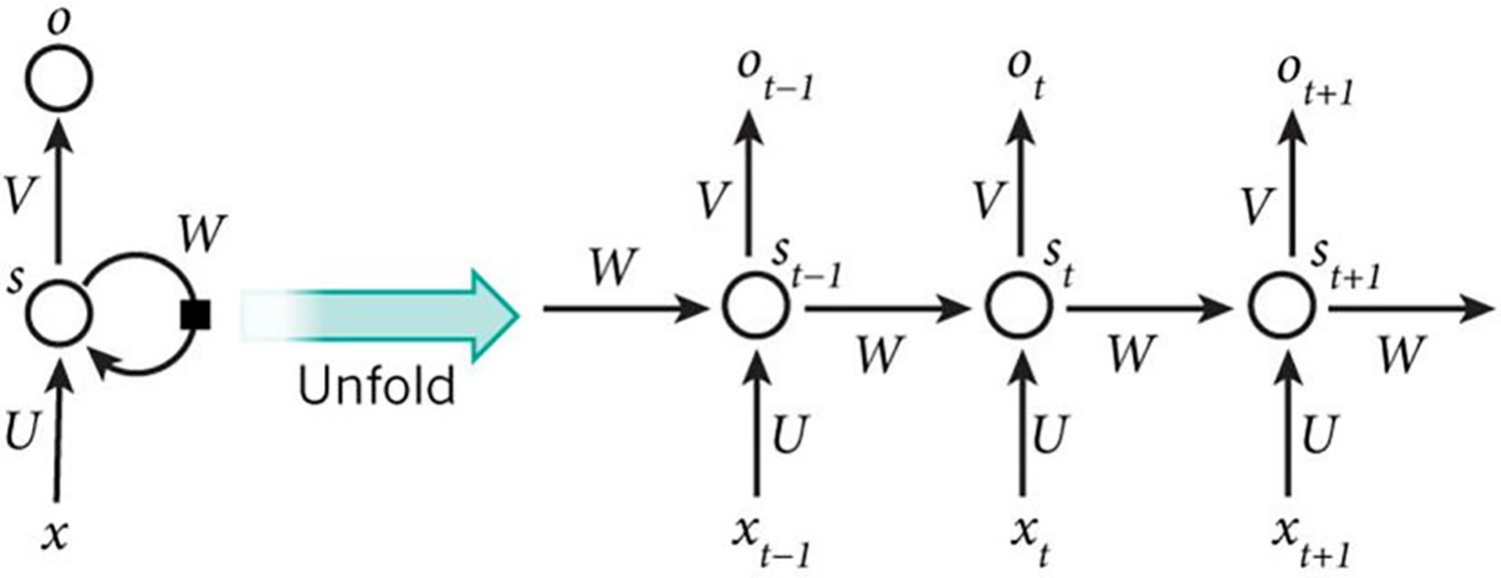
The architecture diagram of recurrent neural network.

In an RNN, the output of a neuron in the current step can directly affect its input in the next step. In other words, for the i-th layer neuron in an RNN network, its input at time t includes the output from the i-1-th layer neuron at time t-1. It also includes the input at time t-1. In Fig 1, the final result *O*_*t*+1_ of the network at time t+1 is the result of the input at that time combined with all previous inputs. The forward computation of an RNN is unfolded in a time sequence. The network parameters are updated using a time-based back propagation algorithm, which is the most commonly used for training current RNN.

RNN has an internal memory structure design and feed-forward connection, which can effectively process sequence data of any time series. However, for long time series data, the prediction accuracy will decrease, which is significantly lower than the short time series. At the same time, there are also gradient explosion and gradient disappearance, making model training complex and difficult.

### 3.3 Long short-term memory neural network

Although the RNN model has achieved good results in processing time series, the back propagation algorithm used by RNN will cause gradient disappearance or gradient explosion. It cannot solve the long-term dependence. LSTM aims to solve the long-term dependence and overcome the gradient disappearance or gradient explosion of RNN in dealing with time series problems [35].

**Fig 2:**
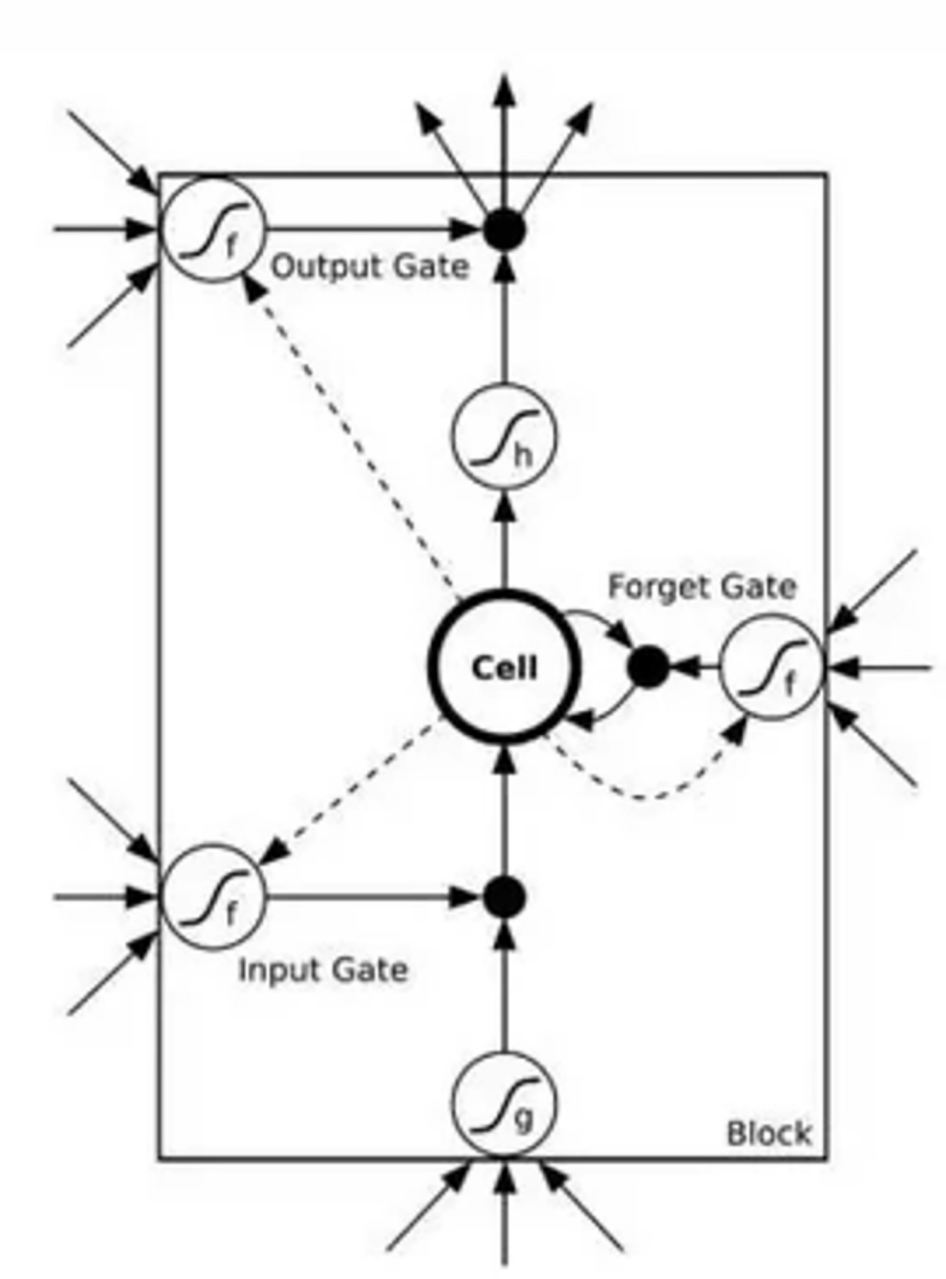
The architecture diagram of long short-term memory.

The LSTM model does not need to debug parameters in a particularly complicated way. It can remember long-term information by default. The core idea of LSTM model structure is a memory unit, which can maintain its state over time. This storage unit, together with nonlinear gate units, regulates the inflow and outflow of information. Fig 2 shows a typical LSTM model structure. It mainly consists of storage unit states of the LSTM network using these three gate structures, namely input gates, forget gates, and output gates [36].

The information forgot in the memory unit is determined by the Sigmoid layer of the forget gate. This layer takes the input *x*_*t*_ of the current layer as input value and the output from the previous layer *h*_*t*−1_. The output of the memory unit state at time t is given by:

$$f_{t}=\sigma (W^{\left(f\right)}x_{t}+U^{\left(f\right)}h_{t-1}+b^{\left(f\right)})$$ (5)

Storing information in memory units primarily consists of two parts. (a) The result *i*_*t*_ from the Sigmoid layer of the input gate, which serves as the information to be updated. (b) A newly created vector *u*_*t*_ from the tanh layer, which is added to the state of memory unit. To forget information, the old memory unit state *f*_*t*_ is multiplied by *c*_*t*−1_. Then the sum of the new candidate information *i*_*t*_·*u*_*t*_ is generated to update the memory unit state.


$$i_{t}=\sigma (W^{\left(i\right)}x_{t}+U^{\left(i\right)}h_{t-1}+b^{\left(i\right)})$$ (6)

$$u_{t}=\tanh(W^{\left(u\right)}x_{t}+U^{\left(u\right)}h_{t-1}+b^{\left(u\right)})$$ (7)

$$c_{t}=i_{t}⊗u_{t}+f_{t}⊗c_{t-1}$$ (8)

The output information is determined by the output gate. Firstly, the Sigmoid layer is used to determine partial information to be output from the memory unit state. Then the memory unit state is processed by tanh. The output value is the product of these two parts of information.


$$c_{t}=i_{t}⊗u_{t}+f_{t}⊗c_{t-1}$$ (9)

$$h_{t}=o_{t}⊗\tanh(c_{t})$$ (10)

The LSTM neural network model has high prediction accuracy, strong learning ability, high robustness, and high fault tolerance. It can fully fit complex nonlinear functional relationships. It is particularly good at dealing with time series problems with long-term dependency characteristics. The LSTM neural network model is more in line with the demand for predicting long time interval time series data in the stock market [37, 38].

### 3.4 EEMD-LSTM early warning model

**Fig 3:**
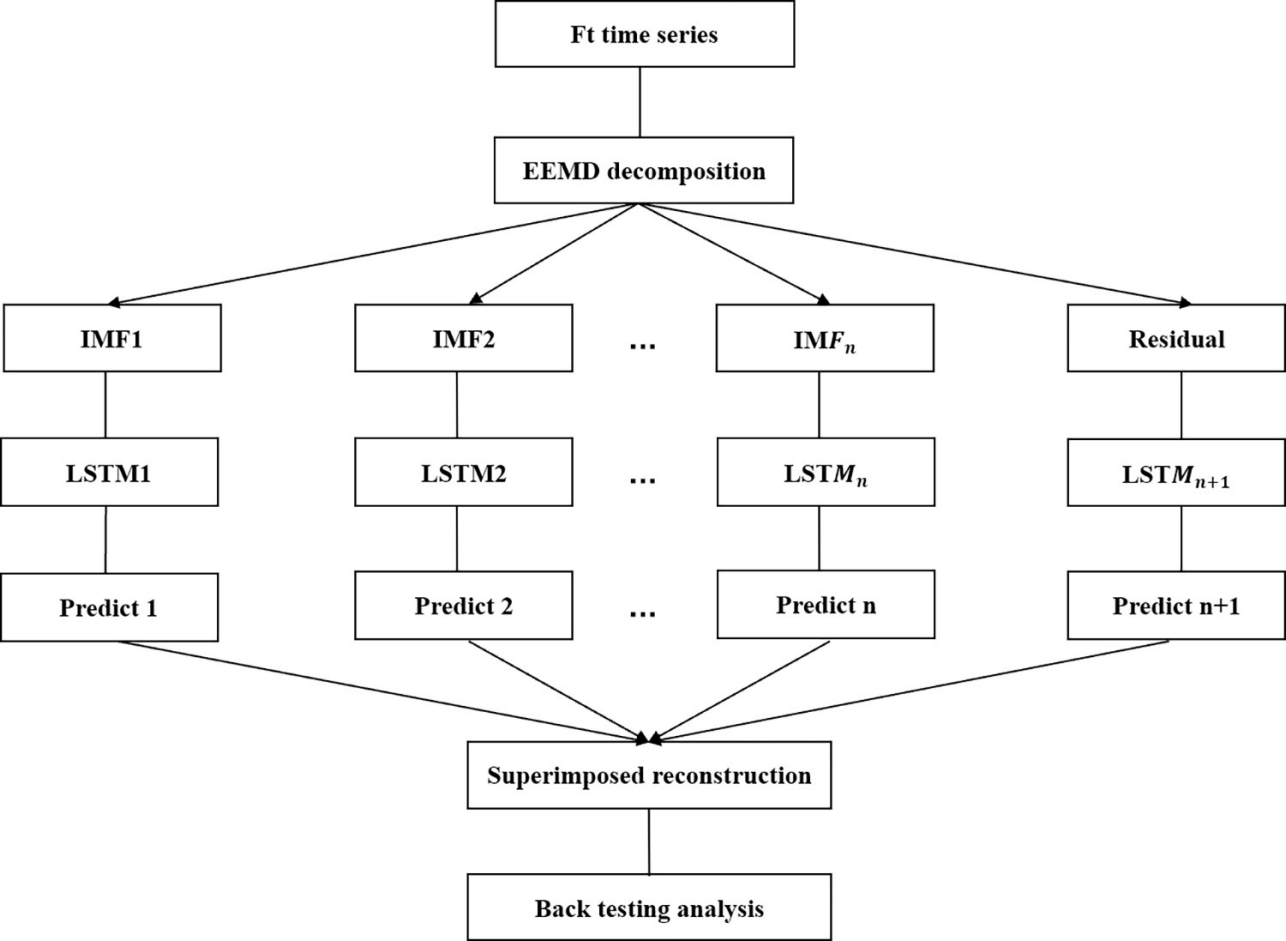
EEMD-LSTM modeling flowchart.

The comprehensive systemic risk index proposed in this paper is a typical nonlinear complex sequence. The predictive efficiency of simple traditional econometric models is limited. EEMD technology has outstanding advantages in processing non-stationary time series. Meanwhile, LSTM also has significant advantages in characterizing long-term memory of time series [39]. Therefore, this paper refers to the TEI@I methodology for complex system research. Then a EEMD-LSTM model for systemic risk warnings of the stock market is established. The detailed modeling process is shown in Fig 3.

1. Using EEMD technology to decompose the comprehensive systemic risk index *F*_*t*_ in the stock market, thus gaining IMF components and residual terms.
2. Normalizing IMF components and matching corresponding training and testing sample data.
3. Training different IMF components by LSTM networks, so as to obtain individual predictive value for each IMF component.
4. Integrating all the prediction results of each IMF sub-sequence, and conducting ensemble prediction of *F*_*t*_ index and back-testing [40].

The computation of the EEMD decomposition of the new sequence in the first step is shown in Eq (11).


$$x*(t)=x(t)+\sum_{i=1}^{N}\varepsilon_{n}*z_{i}(t),z_{i}(t)∼N(0,1)$$ (11)

In Eq (11), *ε*_*n*_ is the standard deviation, x*(*t*) is the new sequence in time with the added noise signal, *N* is the number of times the noise is added, and A is the noise. After completing the expression of the new sequence continue the EMD decomposition and calculate its IMF value. The EMD decomposition with added noise is then repeated until all IMF functions and residual functions are computed. These data features are extracted and input into LSTM for prediction. The normalization of sequence data prediction is shown in Eq (12).


$$z_{i,j}=(x_{i,j}-\min(x_{j}))/(\max(x_{j})-\min(x_{j}))$$ (12)

In Eq (12), min(*x*_*j*_) and max(*x*_*j*_) denote the minimum and maximum values of the sequence of *x*_*j*_, and *z*_*i*,*j*_ denotes the time series difference. The equation for inverse normalization is shown in Eq (13).


$${\hat{y}}_{i`}=(y_{i`}-close\_preice_{\min})/(close\_price_{\max}-close\_price_{\min})$$ (13)

In Eq (13), *y*_*i*`_ denotes the normalized result predicted by the model and ${\hat{y}}_{i`}$ denotes the inverse normalized value.*close*_*price*_max_ and *close*_*price*_min_ denote the closing maximum and minimum values, respectively.

## 4. Indicator construction

### 4.1 Indicator selection and processing

In this paper, based on the characteristics of systemic risk and the actual development of the Chinese stock market, a comprehensive systemic risk index is established from three perspectives: macroeconomic operations, market cross-infection, and the stock market itself. Furthermore, the behavioral finance theory is also taken into consideration in building up the model. Actually, the rationality of stock market participants is limited. The fluctuations of investor sentiment have effects on market volatility, which is particularly evident in the Chinese stock market.

On the basis of drawing on domestic and foreign research results, a total of 35 systemic risk indicators for the stock market are selected. An early warning indicator system for the Chinese stock market is constructed [41–43]. The specific indicators and their economic implications are shown in Table 2. In addition, considering that the selected indicators can comprehensively reflect different aspects of systemic risk and are in line with the specifics of the Chinese stock market, there are also investor sentiment fluctuations that have an impact on market volatility. Therefore, the indicator that can reflect investor sentiment is selected, i.e., the Composite Index of Investor Sentiment of Chinese Stock Market. Its calculation equation is as follows:

CICSI=0.231DCEF+0.224TURN+0.257IPON+0.322IPOR+0.268CCI+0.405NI (14)

ISI=0.64NA+0.521TURN+0.229CCI+0.351DCEF+0.227NIPO+0.463RIPO (15)

OISI=0.72NA−0.163DCLOSE+0.451DCEF+0.761POR+0.662CPI−0.51TUR (16)

In Eq (14), CICIS represents the composite index of investor sentiment in China’s stock market, DCEF represents the fund’s discount rate, with a coefficient value of 0.231. TURN represents the trading volume in the previous month, with a coefficient value of 0.224. IPON is the number of IPOs, with a coefficient value of 0.257. IPOR is the weighted-average yield of the first day of new shares outstanding in the month, with a coefficient value of 0.322. CCI is the number of new accounts opened last month, with a coefficient value of 0.268. CCI is the number of new investors who opened new accounts last month, with a coefficient value of 0.268. NIA is the Consumer Confidence Index, with a coefficient value of 0.405. In Eq (15), NA, TURN, CCI, DCEF, NIPO, and RIPO stand for last month’s average closed-end fund discount rate, average return on the first day of IPOs, the number of new shares, the number of new accounts opened, the market turnover rate, and the consumer confidence rate last month, respectively. The values of the coefficients are 0.64, 0.521, 0.229, 0.351, 0.227, and 0.463 for last month’s Consumer Confidence Index (CCI), and the values of the coefficients are 0.64, 0.521, 0.229, 0.351, 0.227, and 0.463 for each coefficient, respectively.In Eq (16), the NA, DCLOSE, DCEF, POR, CPI, and TUR stand for the number of new account openings, the return of the CSI Composite Index, the discount rate of the closed-end funds, and the weighted average liquidity return on the new stocks respectively, consumer price index and the first-order difference of the turnover rate. The values of the coefficients are 0.72, 0.163, 0.451, 0.761, 0.662, 0.51, and 0.501, respectively.

**Table 2:** Early warning indicators of systemic risk in stock market.

Perspectives	Secondary indicators	No.	Names of indicators	Economic meaning
Macroeconomic operation	Economic operation	M1.1	M2 year-on-year growth rate /M1 year-on-year growth rate	Stronger risk resistance when M1 growth surpasses M2 growth
		M1.2	M2 year-on-year growth rate /GDP year-on-year growth rate	Weaker risk resistance when M2 growth exceeds GDP growth
		M1.3	CPI year-on-year growth rate	Higher inflation rates indicate greater risk
		M1.4	PPI year-on-year growth rate	Higher corporate profits signify stronger risk resistance
		M1.5	PMI index	Higher values suggest relatively lower risk
		M1.6	Economic policy uncertainty	Increased uncertainty implies weaker risk resistance
Market cross-infection	Financial institutions	I1.1	Loan-to-deposit ratio of financial institutions	Higher ratios indicate lower bank liquidity and weaker risk resistance
		I1.2	Loan year-on-year growth rate /GDP year-on-year growth rate	Risk increases when loan growth exceeds GDP growth
		I1.3	Short-term loan balance year-on-year growth rate /GDP year-on-year growth rate	Risk rises when short-term loan balance growth outpaces GDP growth
		I1.4	Medium-and long-term loans/total loans	Higher ratios signify lower asset liquidity and weaker risk resistance
		I1.5	Non-performing loan ratio	Higher ratios signify greater financial system risk
		I1.6	1-week and 1-year shibor term spreads	Wider spreads suggest greater risk
		I1.7	Shibor-libor 1-week spread	Wider spreads suggest greater risk
	Bond market	I2.1	China bond composite index (gross) wealth index year-on-year	Investors tend to buy bond assets and sell equities during financial risk, leading to increased overall bond returns
		I2.2	5-year treasury bond and 3-month treasury bond	Wider spreads indicate greater risk
			Yield to maturity spread	
		I2.3	Interest rate on 10-year treasury bonds	Higher interest rates imply greater risk
	Foreign exchange market	I3.1	Year-on-year growth rate of foreign exchange holdings	Higher reserves imply stronger risk resistance
		I3.2	Real effective exchange rate index	Significant currency depreciation is a primary indicator of financial crises
		I3.3	Year-on-year growth rate of foreign exchange reserves	Higher reserves imply stronger risk resistance
		I3.4	Year-on-year growth rate in the total value of imports and exports	Faster growth suggests stronger risk resistance
		I3.5	Year-on-year growth rate in export value for the month	Faster export growth suggests stronger risk resistance
		I3.6	Year-on-year growth rate in import value for the month	Faster import growth suggests weaker risk resistance
	Real estate market	I4.1	Completed investment in real estate development	Greater investment indicates higher risk
		I4.2	Commercial housing sales: cumulative year-on-year growth rate	Higher sales volume indicates higher risk
		I4.3	National real estate climate index	Higher index indicate lower risk
Stock market	Investor sentiment	S1.1	CICSI sentiment index	Sentiment indicators reflects investor psychology and can trigger irrational market behavior
		S1.2	ISI sentiment index	Sentiment indicators reflects investor psychology and can trigger irrational market behavior
		S1.3	OISI sentiment index	Sentiment indicators reflects investor psychology and can trigger irrational market behavior
	Stock market operation	S2.1	Shanghai composite index yield	Higher returns indicate greater risk
		S2.2	Volatility of the Shanghai Composite Index	Higher volatility implies greater risk
		S2.3	Year-on-year growth rate of total market value of listed companies	Faster growth indicates stronger risk resistance
		S2.4	Year-on-year growth rate in stock market turnover	Faster growth indicates stronger risk resistance

		S2.5	Year-on-year growth rate of stock market trading volume	Faster growth indicates stronger risk resistance
		S2.6	Average price to earnings (PE) ratio	Higher values imply relatively greater risk
		S2.7	Average price to book (PB) ratio	Higher values imply relatively lower risk

Considering effectiveness and availability, the warning indicators selected in this paper are all data from January 2007 and September 2019. The reason for this is that this period covers the key global financial crisis and the subsequent economic recovery phase, which is important for the study of systemic risk. The financial crisis of 2007 had a profound impact on the global stock market, while the following years witnessed market volatility and recovery. In addition, this time period covers an important stage of development of the Chinese stock market, making the data not only highly representative, but also able to provide important insights into the long-term trends and cyclical fluctuations of the market, which can help construct more comprehensive and accurate risk warning models. Monthly data are collected from major resources websites, including the Wind database, CSMAR database, National Bureau of Statistics, People’s Bank of China, State Administration of Foreign Exchange, and China Bond Information Network. Among them, quarterly data such as GDP and non-performing loan ratio are converted using interpolation.

Next, these preliminary indicators are screened in two stages. In the first stage, indicators with weak correlation of the Shanghai Composite Index are removed based on the correlation coefficient. 10 irrelevant indicators are eliminated, and 25 indicators are ultimately selected. In the second stage, the selected indicators are used to build a correlation coefficient matrix. Indicators with a correlation coefficient greater than 0.7 indicate higher similarity. Then indicators with lower coefficient of variation are removed. Finally, 14 indicators are selected for further research, as shown in Table 3.

**Table 3:** Early warning indicators of systemic risk in stock market after screening.

No.	Name of indicators
M1.1	M2 year-on-year growth rate /M1 year-on-year growth rate
M1.2	M2 year-on-year growth rate /GDP year-on-year growth rate
M1.5	PMI index
I1.5	Non-performing loan ratio
I1.7	Shibor-libor 1 week spread
I2.1	China Bond Composite Index (gross) wealth index year-on-year
I2.3	10-year treasury bond rate
I3.1	Year-on-year growth rate of foreign exchange holdings
I3.2	Real effective exchange rate index
I4.3	Real estate climate index
S1.3	OISI sentiment indicator
S2.1	Shanghai Composite Index yield
S2.2	Volatility of the Shanghai Composite Index
S2.4	Year-on-year growth rate in stock market turnover
S2.5	Year-on-year growth rate in stock market trading volume

### 4.2 Principal component analysis

Considering the interpretability of the time series and in order to preserve the initial series information as much as possible, the study used PCA to factor analyze the monthly cross-sectional data of the sample group [44]. Before factor analysis, it is necessary to eliminate the influence of indicator dimensions and orders of magnitude. In the neural network modeling, the convergence of the network is trained. Therefore, the Z-SCORE standardization method is used to normalize the data under each indicator in the sample. In addition, considering that the study data covers the period from January 2007 to September 2019, it indicates that the data set is of longer time span. Therefore, the setting of the synchronization period in the subsequent stock market risk analysis is set to be month to meet the volatility and dynamic nature of the stock market data. In the correlation test of original variables, the KMO statistical value is 0.665. The *P* value of Bartlett’s spherical test is less than 0.01. The spherical hypothesis is rejected. There is a correlation between the original variables [45]. Combined with the KMO statistical value and Bartlett’s spherical test result, the data is suitable for factor analysis. According to the principle that the feature value is greater than 1, five principal component factors are extracted. The cumulative variance contribution rate reaches 85.896%. Principal component factors can reflect most of the information of early warning indicators and can replace the original indicators for early warning analysis. Using PCA to construct a composite systematic risk index for the Chinese stock market first involves selecting and standardizing multidimensional indicators related to systematic risk (e.g., market volatility, trading volume, macroeconomic data, etc.). Next, the PCA methodology is applied to extract the main directions of change of these indicators, i.e., the principal components, in order to capture the key risk factors of the market. Based on these principal components and their explained variance ratios, a composite index is constructed to reflect the overall risk profile of the Chinese stock market. Historical data backtesting and continuous adjustment are used to ensure that the index accurately reflects the systematic risk of the market. The results are shown in Table 4.

**Table 4:** Principal component eigenvalues and contribution rates.

Principal component	Eigenvalue	Contribution rate	Cumulative contribution rate
1	3.327	23.879	23.879
2	2.889	20.735	44.614
3	2.819	20.231	64.845
4	1.674	12.013	76.858
5	1.259	9.038	85.896

Next, the factor scores of each factor can be further obtained through the load factor matrix. Combined with the variance contribution rate of 5 common factors, *F*_1_, *F*_2_, *F*_3_, *F*_4_ and*F*_5_, a composite index of systemic risk in the stock market *F*_*t*_ is established. Then, the financial risk status of each year is determined by dividing the range of *F*_*t*_ values, expressed as follows:

$$F_{t}=\frac{23.879F_{1}+20.735F_{2}+20.231F_{3}+12.013F_{4}+9.038F_{5}}{85.896}$$ (17)

Most of the indicators involved in setting up the composite index are ratios and year-on-year growth rates. A high growth rate of indicator values does not necessarily indicate a safe and sound stock market, and vice versa. Therefore, the study follows the previous approach of scholars such as Xiao et al [46]. This paper determines whether the stock market is facing systemic risk by observing whether the stock market operates steadily and whether the composite index shows violent fluctuations or abnormal values. Specifically, the systemic risk warning line is determined based on the data of the composite index. If the composite index touches or exceeds the systemic risk warning line, it indicates systemic risk.

The “mean ± standard deviation line” and “mean ± two times standard deviation line” are used as the systemic risk warning lines, denoted as W1 line, W1’ line, W2 line, and W2’ line. More specifically, it is considered to trigger a systemic risk warning when the systemic risk composite index reaches or exceeds the W1 line, W1’ line, or W2 line, When it reaches or exceeds the W1’ line, it is considered to trigger a “crisis” warning. When it reaches or exceeds the W2 line or W2’ line, it is considered to trigger a “serious crisis” warning.

**Fig 4:**
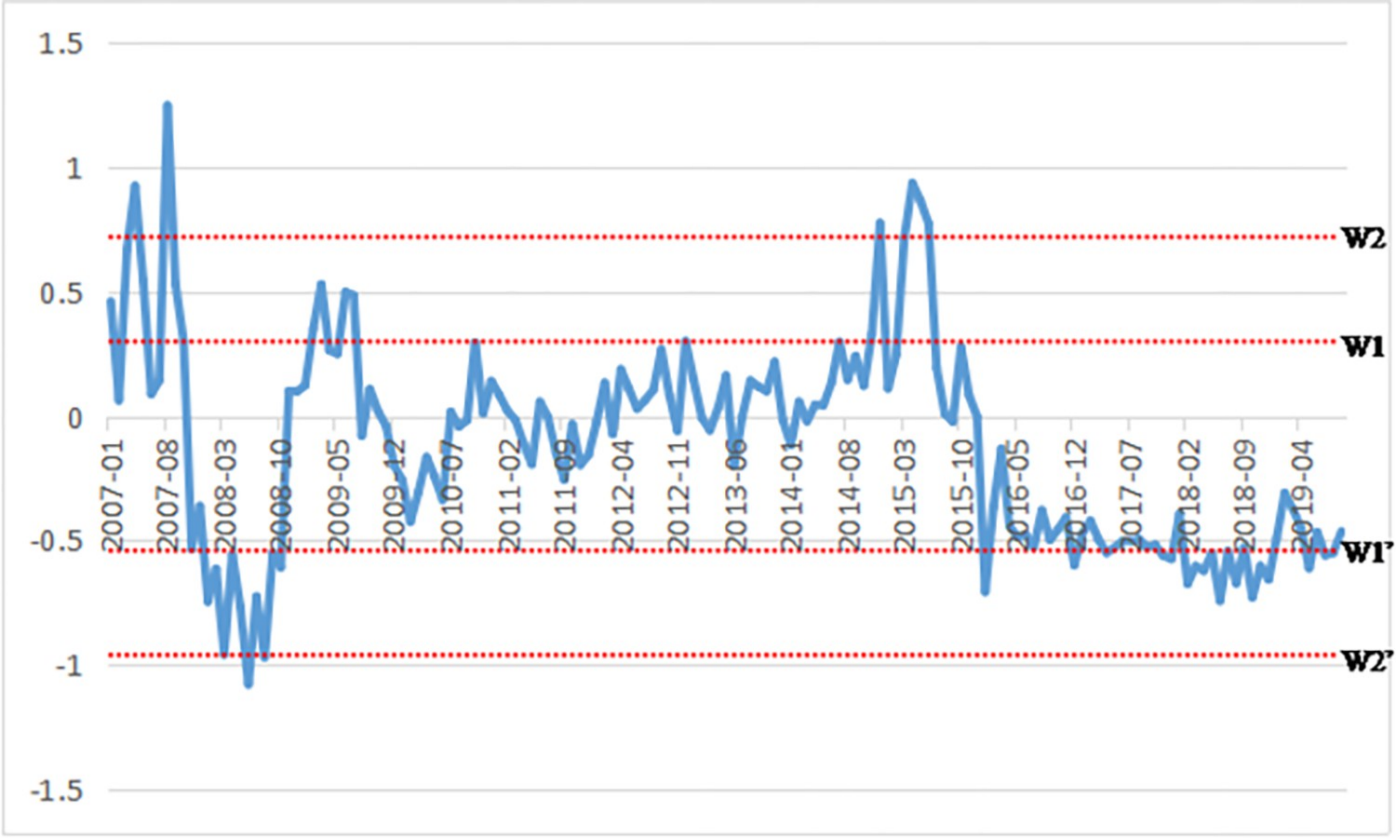
Stock market systemic risk composite index 2007–2019.

The trend chart in Fig 4 shows the composite index of systemic risk in the Chinese stock market from 2007 to 2019. It is evident that abnormal composite index values since 2007 have mainly concentrated in periods from March 2007 to October 2008, from March to June 2015, and from December 2017 to December 2018. During these three periods, the composite index shows continuously abnormal values, with the index exceeding the risk warning line for a long time. Therefore, the systemic risk warning lasts. The systematic risk composite index deviates the most severely in September 2007, June 2008, and May and June 2015, reaching the risk warning lines W2 and W2’. The "serious risk" warning is triggered. The years 2007–2008 and 2015 are two major “stock market disasters” in the history of the Chines stock market. In these periods, the stock market experiences unprecedented surges and crashes, accompanied by volatile fluctuations in stock prices, intensifying market risks. Against the backdrop of slowing economic growth, escalating external uncertainties, and global stock market downturns, stock indices have been continuously declining since 2018, marking a substantial downturn second only to that of 2008. The model results underscore that the Composite Systemic Risk Index triggering early systemic risk warnings are primarily concentrated in the periods of March 2007 to October 2008, March to June 2015, and December 2017 to December 2018. Combined with the above data analysis, it can be seen that the stock market systematic risk composite index built by the PCA methodology is related to the systematic risk of the stock market, mainly because PCA is able to effectively identify and extract the most critical information in the multidimensional risk indicators, so as to construct a comprehensive index reflecting the overall risk status of the market. Several studies in recent years have supported the effectiveness of PCA in constructing stock market systematic risk indicators, such as Alexandridis and Hasan [47]. The risk assessment model developed in this study, founded on macroeconomic performance, cross-market contagion, and inherent stock market dynamics, closely aligns with the economic development and stock market behavior in China. It effectively identifies high-risk junctures, demonstrating strong historical event correlation and interpretability. The application of the Composite Systemic Risk Index and its associated early warning mechanism in risk forecasting exhibits high rationality and effectiveness within the field of risk management.

## 5. Results

### 5.1 Data description and evaluation criteria

In this study, the *F*_*t*_ index generated according to Eq (17) is selected for research purpose, covering data from January 2007 to September 2019. It was divided into training and test sets in the ratio of 8:2 in time, where the training dataset included data from January 2007 to September 2017 The data from this period is used as training for the model for modeling the stock market composite systematic risk index. The test dataset includes data from October 2017 through September 2019. Data from this time period was retained for evaluating the performance of the model and testing the model’s ability to generalize over unseen data. Data processing included normalization of the data under each indicator in the sample using the Z-SCORE normalization method to remove the effects of indicator dimensionality and order of magnitude. Subsequently, five principal component factors were extracted by PCA, which reflect the information of the early warning indicators and can be used to construct a comprehensive systematic risk index of the stock market to achieve the goal of data downscaling and risk analysis.

To assess the predictive performance of various models, this paper employs three evaluation metrics, Root Mean Square Error (RMSE), Mean Absolute Error (MAE), and Mean Absolute Percent Error (MAPE). These metrics are expressed as follows:

$$RMSE=\sqrt{\frac{1}{n}\sum_{i=1}^{n}{\left(y_{i}-{\hat{y}}_{i}\right)}^{2}}$$ (18)

$$MAPE=\frac{1}{n}\sum_{i=1}^{n}\left|\frac{y_{i}-{\hat{y}}_{i}}{y_{i}}\right|\times 100\%$$ (19)

$$MAE=\sum_{i=1}^{n}\left|y_{i}-{\hat{y}}_{i}\right|\times \frac{1}{n}$$ (20)

In Eq (18), *y*_*i*_ is the actual value of the *F*_*t*_ index. ${\hat{y}}_{i}$ is the corresponding predicted output value. *n* is the sample size for the back-testing. *i* is the specific sorting number of the back testing samples. Small values of RMSE, MAPE, and MAE indicate higher prediction accuracy.

### 5.2 EEMD and data normalization

**Fig 5:**
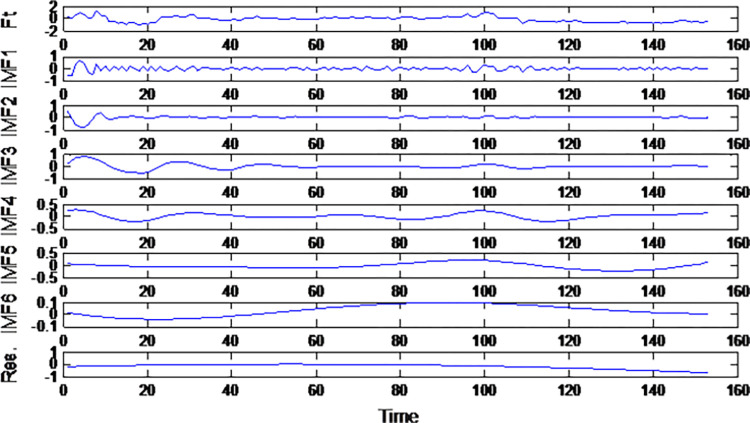
EEMD results of composite systemic risk index in the stock market.

Following the principle of EEMD algorithm, the standard deviation of white noise is set as 0.2. Through 100 cycles of decomposition (NE = 100 times), the original Composite Systemic Risk Index is systemically decomposed. Six IMF components arranged in descending frequency order, as well as the overall residual term Res are obtained. The results of decomposition are illustrated in Fig 5.

It is evident that the decomposed IMF components exhibit a sequential decrease in frequency from top to bottom. This sequencing effectively portrays the changing frequencies and amplitudes characterizing fluctuations in the Composite Systemic Risk Index within the stock market. Notably, these frequency and amplitude dynamics evolve over time. IMF1 encapsulates the high-frequency volatility characteristics of the stock market’s Composite Systemic Risk Index. IMF6 encapsulates the lowest-frequency volatility features of this index. After undergoing EEMD, the fluctuation characteristics of the stock market’s Composite Systemic Risk Index at different scales are distinctly manifest.

Prior to LSTM modeling, it is essential to normalize the data associated with the various IMF components and the remaining residual term Res. This paper adopts the Min-Max standardization method for data normalization, expressed as follows:

$$x^{\prime }=\frac{x-x_{\min}}{x_{\max}-x_{\min}}$$ (21)

In Eq (21), *x*′ is the normalized sub-series component data. *x* is the original value of the sub-series component, *x*_min_ and *x*_max_ denote the minimum and maximum values of each sub-series component.

### 5.3 Analysis of EEMD-LSTM early warning results

To validate the exceptional advantages of the EEMD decomposition integration technology and the predictive efficacy of EEMD-LSTM, this section compares the performance of different models. The performance of models BP, RNN, and LSTM without EEMD decomposition and integration algorithm is compared with the model groups EEMD-BP, EEMD-RNN, and EEMD-L STM combined with EEMD algorithm as a reference. After multiple experimental iterations, when the input dimension of all models is set to 6, it means using data from the first 6 months to predict information for the next month. These models exhibit superior predictive performance. Consequently, the input data dimensions for the aforementioned six models are all set to 6, with their prediction values explicitly expressed as follows:

$${\hat{x}}_{t}=f(x_{t-1},x_{t-2},x_{t-3},x_{t-4},x_{t-5,}x_{t-6})$$ (22)

**Fig 6:**
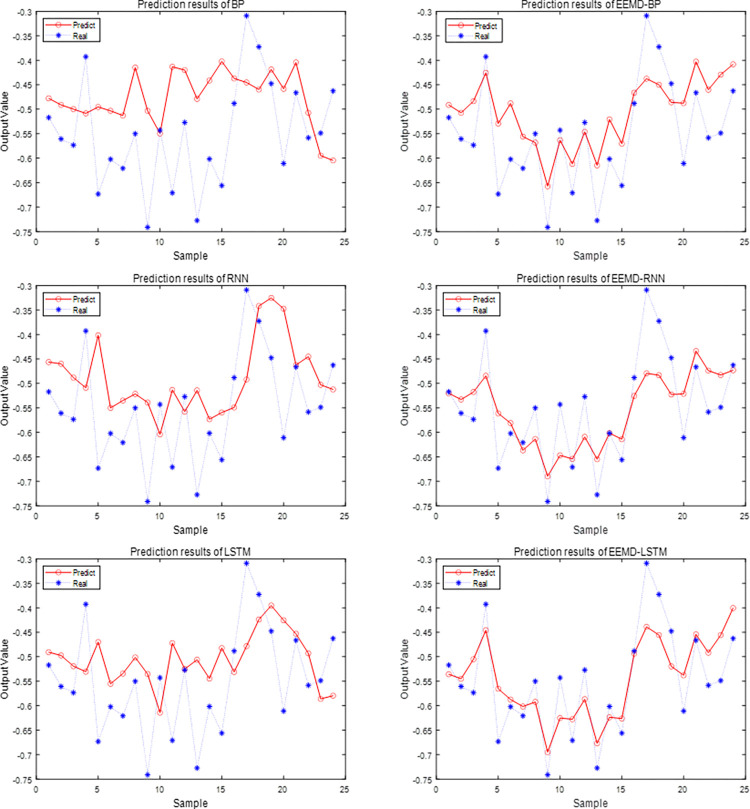
Comparative analysis of test values and predicted values for six machine learning models.

In Eq (22), ${\hat{x}}_{t}$ represents the predicted value of the Composite Systemic Risk Index in the stock market *x*_*t*_ at time *t*. *x*_*t*−1_,…, and, *x*_*t*−6_ represent the values of the Composite Systemic Risk Index in the stock market rolled forward by 6 months at time *t*. The predicted values of the six models on the test samples are illustrated in Fig 6.

The left image in Fig 6 illustrates the predictive performance of machine learning models that do not utilize EEMD integration technology. The right image in Fig 6 depicts the predictive performance of machine learning models integrated with EEMD integration technology. It is evident from the figure that model ensemble combined with the EEMD decomposition algorithm has prediction values that are closer to the true values of the test samples. Notably, the EEMD-LSTM prediction model proposed in this paper demonstrates superior predictive performance. It exhibits the best fit between predicted values and true values compared to the reference models BP, RNN, LSTM and EEMD-BP, EEMD-RNN. The EEMD algorithm effectively enhances the predictive capability of a single model. The predicted value remains within the range of the true value.

In order to further compare the prediction effects and robustness of different models, Table 5 provides a clear overview of the evaluation index values of RMSE, MAPE and MAE, and comprehensively analyzes the prediction performance of the models through three different evaluation indexes.

**Table 5:** Comparison of prediction accuracy among different models.

Prediction model	Error type		
	RMSE	MAE	MAPE
BP	0.1391	0.1187	0.2123
RNN	0.1268	0.1027	0.1874
LSTM	0.1189*	0.0972*	0.1771*
EEMD-BP	0.0815	0.0721	0.1344
EEMD-RNN	0.0725	0.0599	0.1225
EEMD-LSTM	0.0619**	0.0530**	0.1058**

Note: * denotes the optimal performance among models without EEMD technology. ** denotes the optimal performance among models integrated with EEMD technology.

From Table 5, all integrated models incorporating EEMD integration technology exhibit substantially lower RMSE, MAE, and MAPE values compared to their non-integrated models. Their predictive performance significantly surpasses that of models without EEMD integration technology, thereby underscoring the superiority of the decomposition integration technology. It originates from the TEI@I complex system research methodology.

Through the EEMD signal decomposition method, the complexity of time series can be effectively reduced by decomposing the original series into IMF time series with lower complexity. This simplification facilitates the application of machine learning algorithms to enhance the predictive accuracy of each IMF series. Consequently, the prediction integration from each IMF component can enhance the overall series prediction performance. Additionally, considering the dependence of time series and long-term memory characteristics, the LSTM algorithm outperforms the BP and RNN algorithms, achieving more favorable RMSE, MAE, and MAPE values and yielding superior predictive results. The combination of EEMD technology and LSTM algorithm embodied in EEMD-LSTM model is always superior to other combined models in all three evaluation indexes, which shows that it can effectively improve the prediction accuracy and has the robustness of prediction performance compared with other models.

## 6. Conclusions and discussion

Global regulators have been concerned about systemic risk in the stock market, but existing research lacks portfolio models describing complex nonlinear interrelationships. This study fills this gap and constructs a stock market systemic risk indicator system suitable for China’s national conditions. From the three perspectives of macroeconomics, market contagion and stock market characteristics, the TEI@I complex system research methodology is introduced and the EEMD-LSTM model is proposed. The original sequence is decomposed by EEMD algorithm, and then artificial intelligence algorithms such as LSTM are used for forecasting and modeling, and finally the output results are integrated. The innovation of the study is that it not only focuses on traditional market and economic indicators, but also takes into account non-traditional factors such as investor sentiment to capture market risk in a more comprehensive manner. The study highlights the economic impact on Chinese stock market investors, regulators and market stability in the results. By synthesizing a stock market systematic risk index constructed from 35 indicators, the study effectively identifies high-risk points in time. The introduction of a signal decomposition algorithm (EEMD) further improves the early warning effect and helps regulators monitor the market more effectively. Compared with the benchmark model, the EEMD-LSTM model performs better in predicting stock market systematic risk, which has an important economic rationalization impact on enhancing market stability and investor confidence. In addition, the model performs well in identifying high-risk periods in the market, sending out warning signals in advance, which provides investors with a valuable basis for decision-making. It has relatively high predictive accuracy and reduces false positives and false negatives. These findings provide scientific decision support for stock market stakeholders and have positive implications for promoting healthy economic growth and long-term market stability. By comparing with important periods in the actual market, the study allows for a more comprehensive assessment of the model’s performance and provides guidance for future improvements. At the heart of the study lies a comprehensive framework that combines multi-dimensional indicator analysis and advanced forecasting techniques, which not only improves the accuracy of predicting stock market risk, but also provides stock market regulators and investors with a more comprehensive and in-depth tool for assessing market risk.

The study differs from the general literature in four ways. First, the risk factors need to be assessed and updated periodically and change with the market environment. Second, this study focuses on stock market forecasting for the Chinese market, taking into account market differences in different regions. Third, the difference in the accuracy of different risk warning methods may be related to factors such as data quality, model parameters, and market environment. The study finds that there is a significant correlation between the stock market and the macroeconomy, which is consistent with previous literature. Fourth, market sentiment and policy changes have a significant impact on stock market volatility. Fluctuations in investor sentiment, such as optimism or pessimism, as well as adjustments in economic and financial policies set by the government, directly affect the volatility of stock prices, and this uncertainty causes investors to adjust their strategies. The above findings are consistent with the results of existing studies. Although the EEMD method improves the modal aliasing problem in EMD, its own limitations may affect the reliability of the EEMD-LSTM stock market systematic risk early warning model. Specifically, the white noise introduced by EEMD may interfere with the signal and affect the prediction accuracy, while the stochastic nature of the algorithm may lead to instability in the decomposition results. In addition, the relatively high computational complexity of the EEMD algorithm limits its application in resource-limited environments. Therefore, although EEMD-LSTM performs well with stock market data, these limitations need to be taken into account when assessing its overall reliability.

Considering the limitations of the EEMD approach, future work should focus on improving the algorithm and enhancing model stability. This includes optimizing EEMD to reduce noise interference, improving the accuracy and repeatability of the decomposition, exploring more efficient computational methods to adapt to different environments, and incorporating advanced forecasting techniques, such as deep learning, to enhance the model’s adaptability and predictive ability to market dynamics. Such efforts will enhance the overall reliability of the EEMD-LSTM model in stock market risk warning. Overall, the study provides a scientific research methodology for systemic risk early warning in the Chinese stock market, which provides a useful supplement to stock market risk management theories and methods, and the empirical results and early warning are of great significance to investors and regulators.
